# Tocotrienols Influence Body Weight Gain and Brain Protein Expression in Long-Term High-Fat Diet-Treated Mice

**DOI:** 10.3390/ijms21124533

**Published:** 2020-06-25

**Authors:** Yugo Kato, Yoshinori Aoki, Koji Fukui

**Affiliations:** 1 Molecular Cell Biology Laboratory, Department of Functional Control Systems, Graduate School of Engineering and Science, Shibaura Institute of Technology, Fukasaku 307, Minuma-ku, Saitama 337–8570, Japan; nb19102@shibaura-it.ac.jp; 2 Mitsubishi-Chemical Foods Corporation, Marunouchi 1–1–1, Chiyoda-ku, Tokyo 100–8251, Japan; aoki.yoshinori.ma@m-chemical.co.jp

**Keywords:** tocotrienols, obesity, brain oxidation, longevity related-protein

## Abstract

Obesity induces serious diseases such as diabetes and cardiovascular disease. It has been reported that obesity increases the risk of cognitive dysfunction. Cognitive dysfunction is a characteristic symptom of Alzheimer’s and Parkinson’s diseases. However, the detailed mechanisms of obesity-induced cognitive dysfunction have not yet been elucidated. The onset and progression of obesity-induced severe secondary diseases such as diabetes, cardiovascular events, and hypertension are deeply connected to oxidative stress. We hypothesized that obesity induces cognitive dysfunction via acceleration of reactive oxygen species (ROS) production. Vitamin E, which is a lipophilic vitamin, has strong antioxidative effects and consists of two groups: tocopherols and tocotrienols. Recently, it has been demonstrated that tocotrienols have strong neuroprotective and anti-obesity effects. In this study, we fed mice a high-fat diet (HFD) from 9 to 14 months of age and assessed the effect of tocotrienols treatment on body weight, brain oxidation levels, and cognitive function. The results revealed that treatment with tocotrienols inhibited body weight gain; further, tocotrienols reached the brain and attenuated oxidation in HFD-treated mice. These results indicate that tocotrienols have anti-obesity effects and inhibit obesity-induced brain oxidation.

## 1. Introduction

The number of people with obesity continues to increase throughout the world [1]. It is well known that the body mass index (BMI, body weight (kg)/square of body height (m)) score is used as the primary index of obesity. The World Health Organization (WHO) defines a BMI score of more than 25 as overweight and more than 30 as obesity (a BMI score above 25 is defined as obesity in Japan.). Obesity is a well-known risk factor of several secondary disorders such as type-2 diabetes, cardiovascular events, and arteriosclerosis; thus, it is important to focus on obesity prevention [2,3]. Recently, several lines of evidence have identified the relationship between obesity and the onset and progression of cognitive dysfunction. It is well known that cognitive dysfunction is a major symptom of Alzheimer’s and Parkinson’s diseases [4,5]. Although it has been reported that the onset and progression of these neurodegenerative disorders are related to oxidative damage regardless of obesity [6], the detailed mechanisms of obesity-related cognitive dysfunction have not yet been elucidated. The oxygen consumption volume of those with obesity is higher than that of normal healthy individuals [7]. Calorie restriction (CR) in mice and rhesus monkeys has shown life span extension via autophagy activation and attenuation of oxidative stress [8,9]. Based on this background information, we propose hypotheses regarding obesity-derived cognitive dysfunction. First, obesity may induce cognitive dysfunction via accumulation of brain oxidation. Second, treatment with antioxidant substances, such as vitamin E (VE), may play a beneficial role in obesity-related oxidative damage.

VE is a lipophilic natural vitamin, and it is present at relatively high levels in palm oil, whole wheat, and rice bran. VE is classified into tocopherols (TOCs) and tocotrienols (T3s), depending on the presence or absence of three double bonds in their side chains. Moreover, each isoform is classified into α-, β-, γ-, and δ-TOCs and T3s by the number and position of methyl groups in the chroman ring. The most well-known biological function of VE is as an antioxidant, and we have already reported that the antioxidant effects of T3s are stronger than those of TOCs in cell culture and animal experiments [10,11]. Recently, it has been reported that T3s have distinctive effects, such as inhibiting hydroxy-3-methylglutaryl-CoA (HMG-CoA) reductase, which is involved with cholesterol synthesis, induction of apoptosis in cancer cells, and neuroprotection [12,13,14]. However, the detailed mechanisms of the neuroprotective effect of T3s during senescence, obesity, and neurodegenerative disorders have not yet been elucidated. It is well known that T3s have anti-obesity effects in vivo and in vitro [15,16,17]. Previously, we investigated anti-obesity effects of T3s in young obesity model mice [18]: T3s significantly inhibited the body weight gain. However, we could not judge whether T3s have growth inhibition or anti-obesity effects. The brain T3s concentrations were tremendously lower than that of TOCs, in spite of 2 months of a T3s-containing diet. In our previous results, it was very difficult to understand whether T3s truly reached the brain and had antioxidative effects or not.

In this study, we fed a high-fat diet (HFD) in the presence or absence of T3s to 9-month-old C57BL/6 male mice for 5 months. We employed 9-month-old age mice, in which the ratio of body weight gain by growth is smaller than in young mice, in order to investigate the anti-obesity effects of T3s. After treatment, we assessed cognitive function, brain oxidation levels, and the antioxidative defense systems. The study purposes were to elucidate 1) the relationship between obesity and cognitive dysfunction via oxidative stress and 2) the effects of T3s on HFD-treated mice.

## 2. Results

### 2.1. Measurement of VE Isoforms in Each Diet

**Figure 1:**
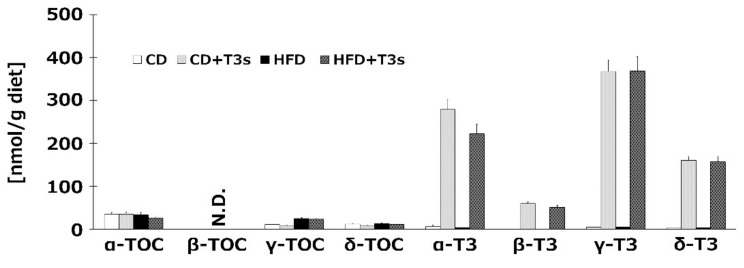
Vitamin E (VE) contents in each diet. VE isoforms in each diet were measured by HPLC-ECD. CD = Control diet (*n* = 4), CD + T3s = CD + tocotrienols (T3) mix (*n* = 4), HFD = high-fat diet (*n* = 4), HFD + T3s = HFD + T3 mix (*n* = 4). Data are expressed as mean ± SE.

To confirm the T3s content in each diet, we measured VE isoforms of all diets before feeding to the animals. The results indicated that sufficient levels of TOCs and T3s were included in each diet, with the exception of β-TOC (Figure 1).

### 2.2. T3s inhibited the Ratio of Body Weight Gain

**Figure 2:**
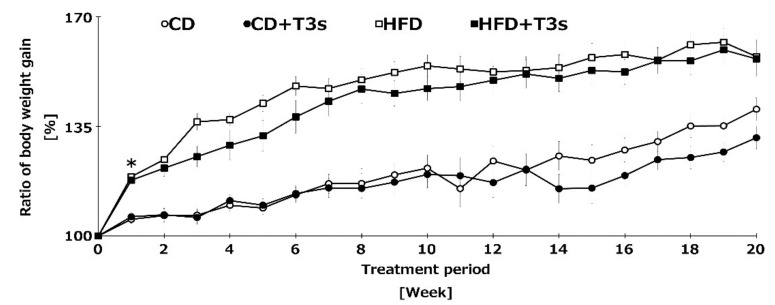
Changes in the ratio of body weight gain of all treatment groups. Body weight was measured once per week for 5 consecutive months. Body weight before feeding of each diet was set to 100%. CD = Control diet (*n* = 10), CD + T3s = CD + T3-\mix (*n* = 10), HFD = high-fat diet (*n* = 10). HFD + T3s = high-fat diet + T3 mix (*n* =10). Data are expressed as mean ± SE, and the timeline shows treatment period. Tukey–Kramer’s method: * *p* < 0.05 treatment period of HFD vs. previous week of HFD.

To clarify the anti-obesity effect of T3s, we measured the body weight of mice once per week. Co-treatment with HFD and T3s tended to inhibit the body weight gain compared to the HFD group until 10 weeks, and the ratio of body weight gain of both control diet (CD) groups were similar until 14 weeks. In addition, the ratio of body weight gain was significantly increased in HFD-treated mice one week after treatment. Co-treatment with HFD and T3s significantly inhibited the body weight gain compared to the HFD group. However, there were no significant differences in the final body weight of HFD-treated mice in the presence or absence of T3s (Figure 2).

### 2.3. T3s Did not Change Food and Calorie Intake

**Figure 3:**
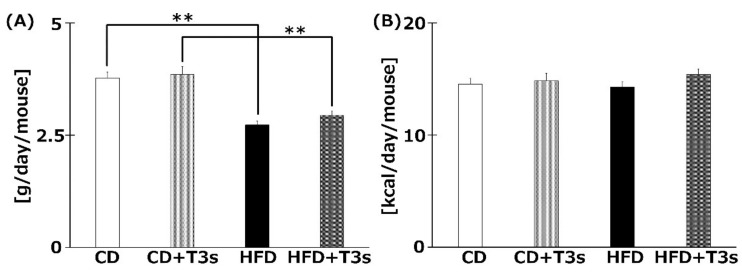
The average food (**A**) and calorie (**B**) intake of each group. Food intake was measured once per week. Calorie intake was calculated from food intake. CD = Control diet (*n* = 10), CD + T3s = CD + T3 mix (*n* = 10), HFD = high-fat diet (*n* = 10), HFD + T3s = HFD + T3 mix (*n* = 10). Data are expressed as mean ± SE. Tukey–Kramer’s method: ** *p* < 0.01.

The food intake (g/day/mouse) is shown in Figure 3A. The food intake was significantly decreased in the HFD-treated group compared to the CD group regardless of the presence of T3s. However, there were no significant differences in calorie intake (kcal/day/mouse) among all groups (Figure 3B).

### 2.4. HFD Did not Induce Cognitive Impairment

**Figure 4:**
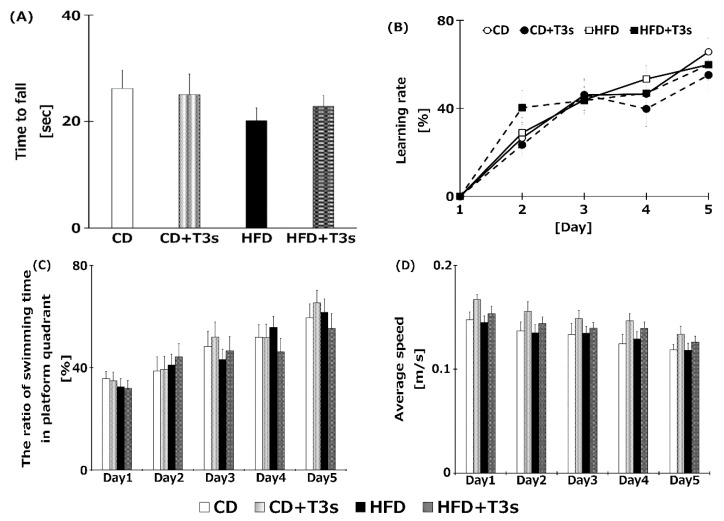
Assessment of cognitive function of each treatment group using the Rota rod test (**A**), the Morris water maze test (**B**), ratio of swimming time in platform quadrant (**C**), and average swimming speed (**D**). Learning ability was calculated from the goal time of each mouse. CD = Control diet (*n* = 14), CD + T3s = CD + T3 mix (*n* = 16), HFD = high-fat diet (*n* = 15), HFD + T3s = high-fat diet + T3 mix (*n* = 13). Data are expressed as mean ± SE.

To clarify the relationship between obesity and cognitive dysfunction, the cognitive function of all mice was measured using the Rota rod and the Morris water maze tests (Figure 4A,B). However, there were no significant differences among the groups.

### 2.5. T3s Reached Target Tissues following Oral Intake

**Figure 5:**
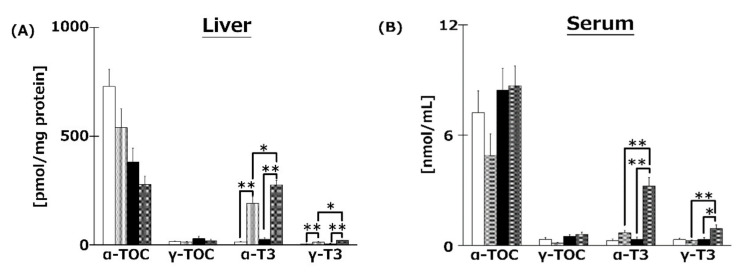

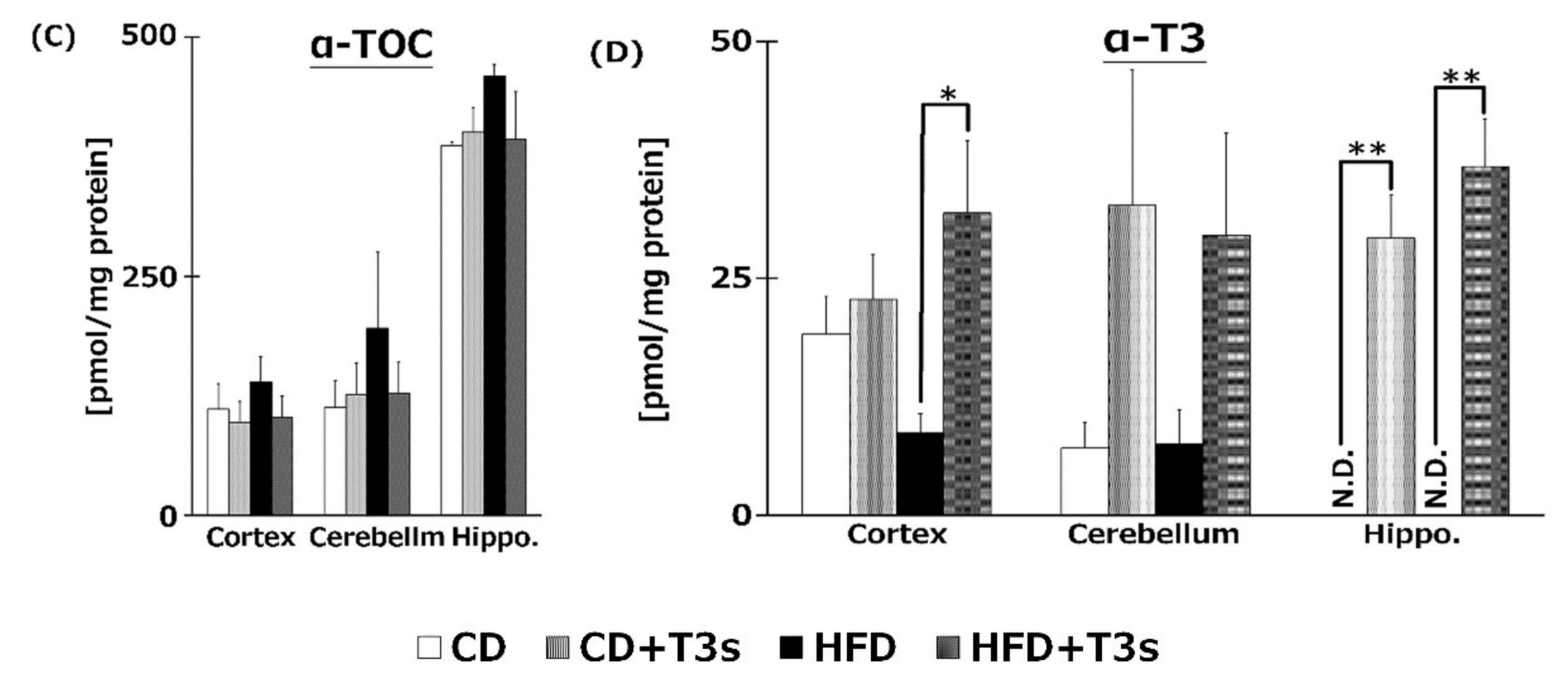
VE content in livers (**A**), serum (**B**), and brains (**C**), (**D**) of mice. T3s were demonstrated to reach the brain. CD = Control diet (*n* = 10), CD + T3s = CD + T3 mix (*n* = 10), HFD = high-fat diet (*n* = 10). HFD + T3s = high-fat diet + T3 mix (*n* = 10). Data are expressed as mean ± SE. Tukey–Kramer’s method: * *p* < 0.05, ** *p* < 0.01.

We measured VE isoforms in the livers (Figure 5A), serum (Figure 5B), and brains (Figure 5C,D) of mice. T3s were found to have reached the livers, serum, and brains from both diets. Both T3 isoforms were significantly increased in the livers and serum of T3s-treated mice. The hippocampal α-T3 levels were significantly increased in both T3s-treated groups.

### 2.6. HFD Did not Change Brain Oxidation Levels

**Figure 6:**
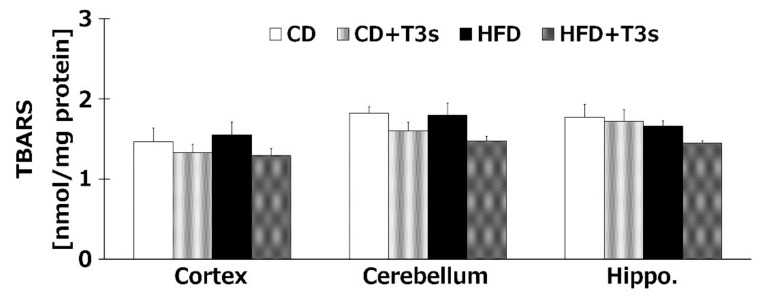
Brain oxidation levels in each treatment group. CD = Control diet (*n* = 10), CD + T3s = CD + T3 mix (*n* = 10), HFD = high-fat diet (*n* = 10), HFD + T3s = high-fat diet + T3 mix (*n* = 10). Data are expressed as mean ± SE.

We investigated the levels of brain oxidation in all treatment groups. Co-treatment with T3s tended to decrease the amount of lipid hydroperoxide products of all brain regions. However, there were no significant differences among all groups (Figure 6).

### 2.7. T3s Enhanced Antioxidative Defense Systems in Brain

**Figure 7:**
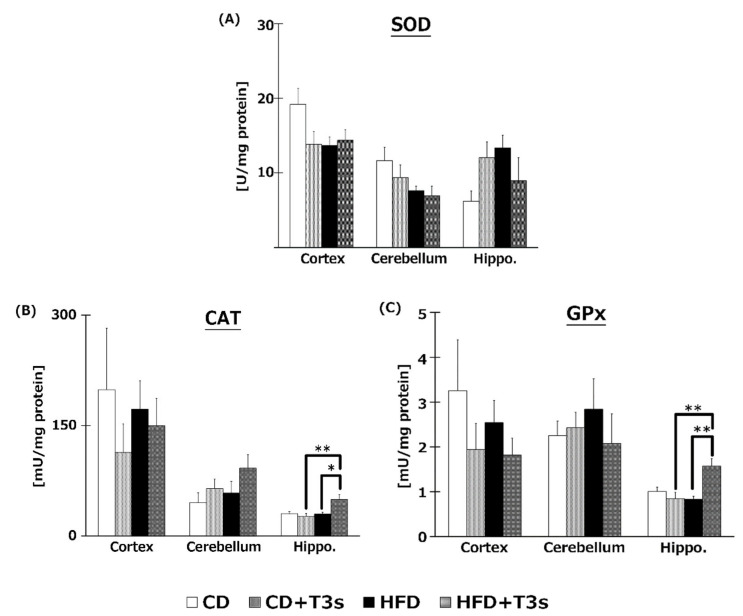
Activities of SOD (**A**), CAT (**B**), and GPx (**C**). CD = Control diet (*n* = 10), CD + T3s = CD + T3 mix (*n* = 10), HFD = high-fat diet (*n* = 10), HFD + T3s = high-fat diet + T3 mix (*n* = 10). Data are expressed as mean ± SE. Tukey–Kramer’s method: * *p* < 0.05, ** *p* < 0.01.

To elucidate the effects of T3s on brain antioxidative systems, we measured SOD (Figure 7A), CAT (Figure 7B), and GPx (Figure 7C) activities. There were no significant differences in SOD activities among all groups. On the other hand, co-treatment with HFD and T3s significantly enhanced CAT and GPx activities in the hippocampus.

### 2.8. Changes in Antioxidative Enzyme Protein Expression

**Figure 8:**
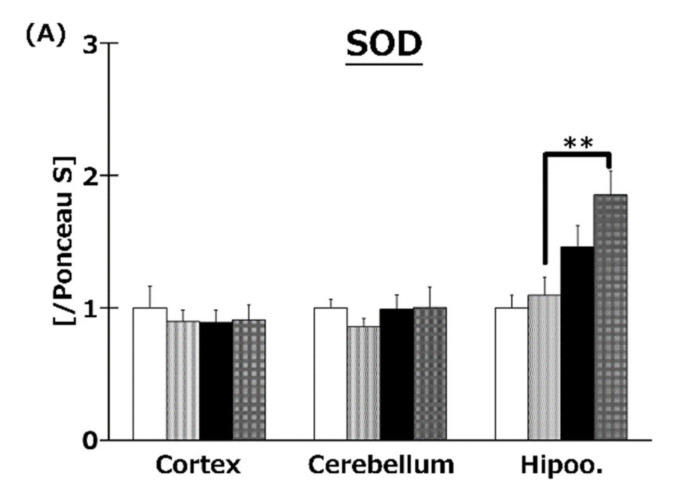

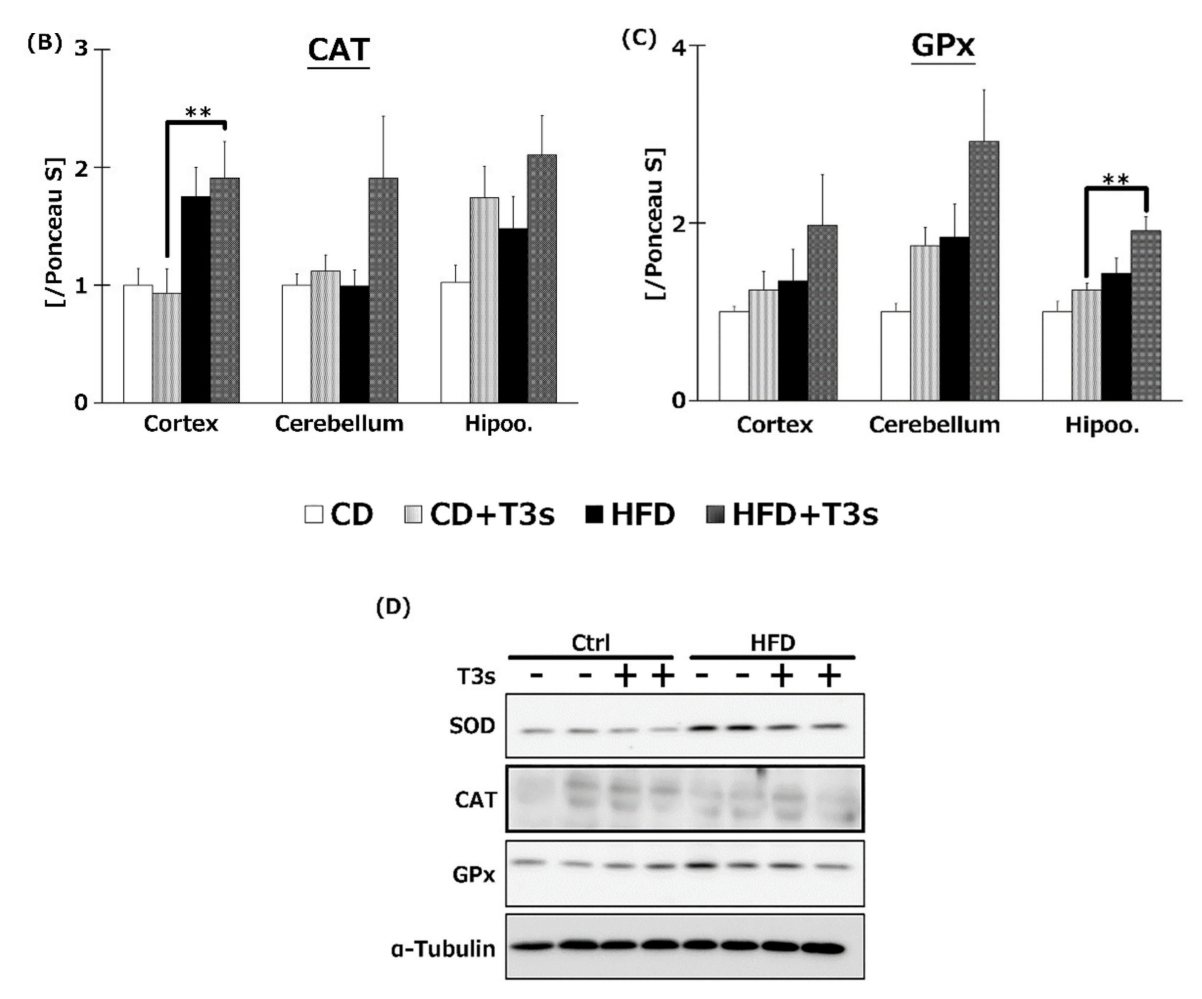
Western blotting analysis of SOD (**A**), CAT (**B**), and GPx (**C**) protein expression. Images of Western blots for each protein examined in hippocampus (**D**). CD = Control diet (*n* = 10), CD + T3s = CD + T3 mix (*n* = 10), HFD = high-fat diet (*n* = 10), HFD + T3s = high-fat diet + T3-mix (*n* = 10). Data are expressed as mean ± SE. Tukey–Kramer’s method: ** *p* < 0.01.

The expression of antioxidative enzyme proteins was measured by Western blotting. Co-treatment with HFD and T3s enhanced SOD1 and GPx1 protein expression in the hippocampus compared to the other three groups (Figure 8A,C). In addition, co-treatment with T3s showed increased CAT protein expression in the cerebral cortex of HFD-treated mice compared to the CD group (Figure 8B).

### 2.9. Changes in the Akt/mTOR Pathway

**Figure 9:**
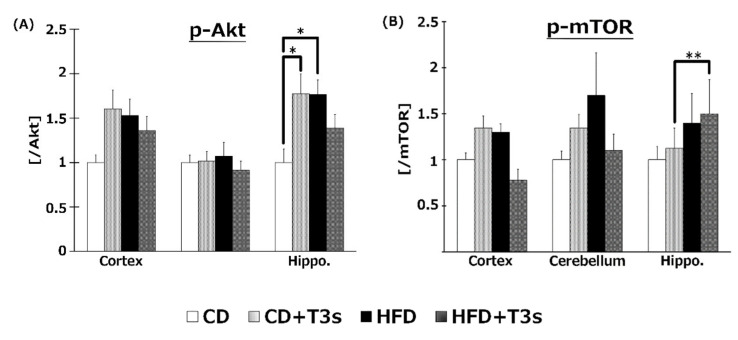

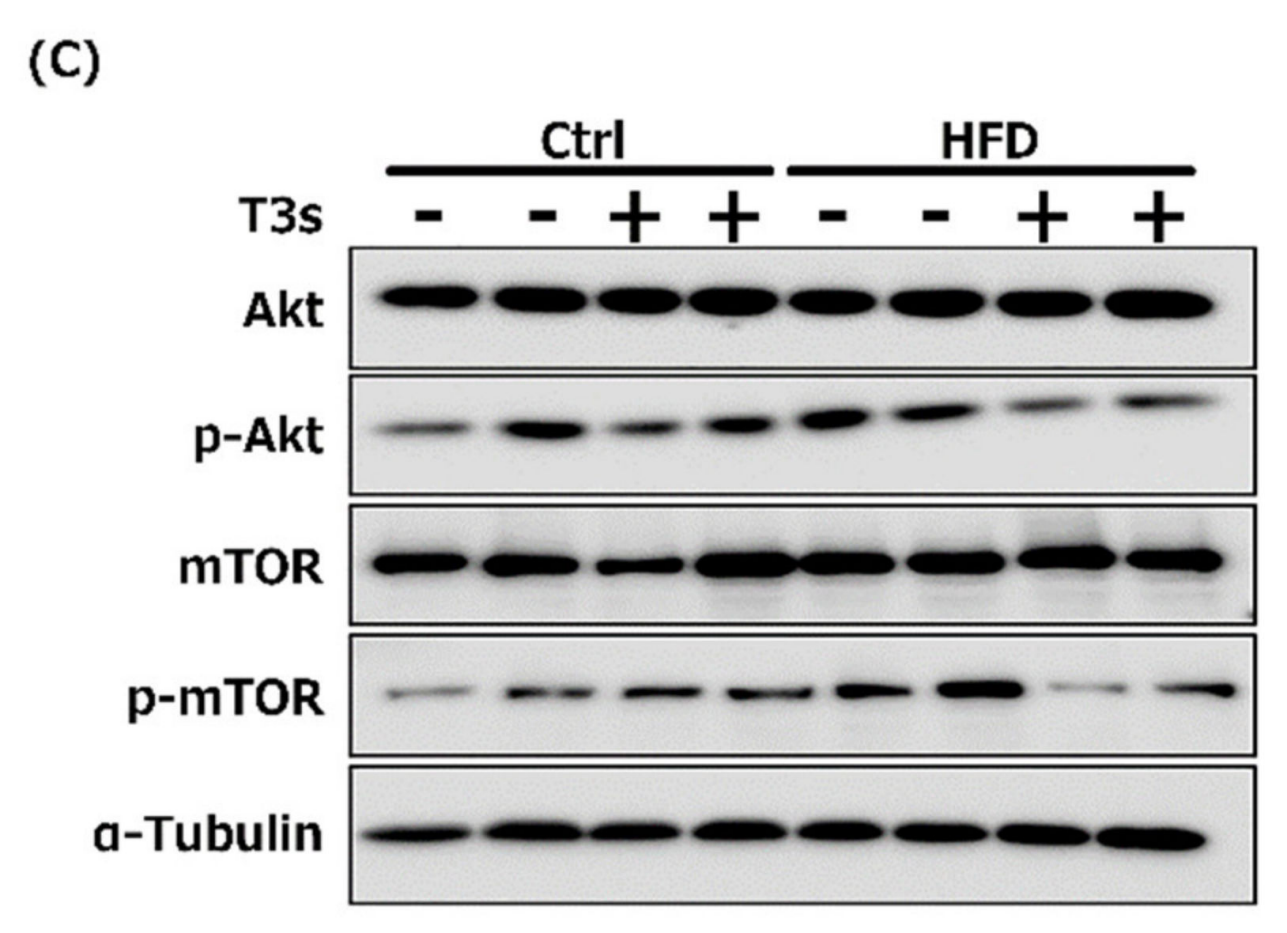
Western blotting analysis of p-Akt (**A**) and p-mTOR (**B**) protein expression. Images of Western blots for each protein examined in cortex (**C**). CD = Control diet (*n* = 10), CD + T3s = CD + T3 mix (*n* = 10), HFD = high-fat diet (*n* = 10), HFD + T3s = high-fat diet + T3-mix (*n* = 10). Data are expressed as mean ± SE. Tukey–Kramer’s method: * *p* < 0.05, ** *p* < 0.01.

To elucidate the effect of T3s on Akt/mTOR signaling, phospho-Akt (Figure 9A) and mTOR (Figure 9B) protein expression were measured by Western blotting. Akt phosphorylation was increased in the hippocampus of CD+T3s and HFD-treated mice. On the other hand, co-treatment with T3s enhanced mTOR phosphorylation in the hippocampus of the HFD-treated group. 

## 3. Discussion

It is well known that T3s, but not TOCs, have strong neuroprotective effect. However, there have been conflicting reports on the anti-obesity effect of T3s. One reason for the inconsistent results may be differences in experimental procedures, such as the treatment period, animal age, etc. In some studies, the treatment period is very short, i.e., only a few weeks, and animals of a young age were employed. This makes judging whether T3s have anti-obesity effects or growth inhibitory effects difficult. To control for these problems, we fed mice T3s over a long-term period, and 9 months of age was set as the starting age. To the best of our knowledge, no study has investigated the effects of T3s on mice for a period of such length and to such an old age as in our study. Additionally, we measured brain oxidation levels in obese mice and investigated the role of T3s in neuroprotection. In this study, long-term treatment with T3s delayed body weight gain in T3s-treated mice (Figure 2). However, the inhibitory effect of body weight gain in HFD-treated mice was not observed in the second half of the treatment period. This result indicates that T3s have a delaying effect but not an arresting effect on body weight gain. The food intake of HFD-treated mice was significantly less than in the CD-treated mice, regardless of the presence or absence of T3s. On the other hand, the calorie intake of all mice did not differ significantly. The reason for the delayed effect of T3s on body weight gain is not due to changes in food intake; thus, a different pathway must be involved. It has already been reported that T3s induce cell cycle arrest and apoptosis via activation of caspace-3 and the AMPK pathway in 3T3-L1 adipocytes [16]. It has been reported that lipid synthesis-related mRNA in differentiated 3T3-L1 cells was modulated by T3s treatment [17]. One mechanism for the delayed weight gain effect of T3s is proposed to be inhibition of fat accumulation. Another reason for the delayed effect of T3s on body weight gain is related to the components of each food. We used HFD and CD in this study. CD is high in nutritive value compared to the normal pellet diet. CD-treated mice showed significantly increased body weight compared to age-matched normal pellet-treated mice (data not shown). CD has been controlled for calculated calories and other nutrients for use as a control diet in experiments using HFD. With long-term treatment of mice with HFD and CD in this experiment, the effect of T3s may be not be able to keep up against the accumulation of fat or the T3s treatment volume may be insufficient. Currently, we are assessing changes in lipid droplets in the livers of HFD-treated mice. T3s were observed to have an anti-obesity effect; however, the effect may not limit weight gain but only delay it.

Cognitive function of mice was measured using the Morris water maze and the Rota rod tests. Treatment with HFD tended to decrease motor coordination ability in the Rota rod test (Figure 4), but not significantly. Additionally, a negative correlation between body weight and time to fall with the Rota rod test was observed. This may be one reason for the impaired motor coordination of the HFD groups. However, the swimming speed in the water maze did not change in all treatment groups; further, there were no significant differences among all groups in the learning ratio of the Morris water maze test. These results showed that cognitive function did not vary significantly under our experimental conditions (treatment period, age of mice, and HFD containing 60% fat). Additionally, long-term HFD treatment did not induce an obvious deficiency in motor coordination. However, there are reports indicating that HFD negatively affects cognitive function. For example, in a study of 20-month-old C57BL/6 mice fed a high fat lard diet for 16 weeks, a significant decline in cognitive function using the T-maze was observed [5]. Furthermore, it was reported that treatment with a high-fat and high-carbohydrate diet for 6 weeks reduced cognitive function using the radial arm water maze in Wistar rats [19]. The research groups reporting obesity-induced cognitive dysfunction assessed cognition using the T-maze or radial arm maze. We did not observe significant differences in cognition in this study; thus, the Morris water maze and the Rota rod test may not be suitable for determining cognitive function in obese mice. Perhaps it would be more appropriate to use a device that records spontaneous movements, such as the open field and the Y maze. In addition, we did a Pearson’s correlation test between cognitive trial results and each biological outcome. However, there were no significant relationships among them. Further investigation is needed to clarification the relationship between obesity and cognitive dysfunction.

In the present study, to clarify the mechanism of the anti-obesity and neuroprotective effects of T3s, we measured the content of each VE isoform in livers, serum, and brains. The liver α- and γ-T3 levels in both T3s-treated groups were significantly higher than those of the untreated groups (Figure 5). Treatment with T3s significantly increased serum α- and γ-T3 levels in HFD-treated mice compared to the untreated groups. However, there were no significant differences between α- and γ-T3 levels of both CD groups in the presence or absence of T3s. It is well known that VE isoforms are absorbed through the small intestine and transported via chylomicrons [20]. Chylomicrons are mainly composed of triglyceride (TG) and cholesterol [21]. The levels of TG and cholesterol tended to increase in the serum of the HFD + T3s group compared to that of the CD + T3s group (data not shown). For the above reasons, serum T3s levels may increase with co-treatment of HFD and T3s. Treatment with T3s in the HFD model increased only the α-T3 level in the brain compared to the untreated group (Figure 5). However, the greatest level of the T3 isoform in the T3s-treated diet was γ-T3 followed by α-T3. α-TOC is selectively bound by the α-tocopherol transfer protein (α-TTP) [22] in the liver, and is involved in transferring α-TOC to other organs. The affinity of α-TTP for α-T3 may be higher than that for γ-T3. Only α-T3 was detected in the brain. 

We confirmed that α-T3 reached the brain. Thus, α-T3 is expected to exert a strong neuroprotective effect via its antioxidant function. T3s showed neuroprotective effects in hydrogen peroxide-treated cultured neuroblastoma cells, namely, inhibition of cell death and protection against neurite degeneration [10]. We expected T3s to show a strong antioxidative effect in this study. Brain oxidation levels were measured using the TBA-method, which is the standard method for determining lipid peroxidation. TBARS values tended to decrease in groups treated with T3s compared to those of the groups not treated with T3s (Figure 6). This phenomenon may be induced by not only the antioxidative effect of T3s but also upregulation of antioxidative systems (Figure 5, Figure 7, and Figure 8). However, there were no significant differences among all groups. This result indicates that progressive brain oxidation was not observed in this experimental model. This suggests that we need to change the experimental methodology, such as the composition of HFD.

To investigate the effect of T3s on antioxidative systems of the brain, antioxidative enzyme activities such as SOD, CAT, and GPx were measured (Figure 7). There were no significant differences in SOD activity in all brain regions. On the other hand, CAT and GPx activities in the hippocampus of the T3s-treated HFD group were significantly increased compared to that of the T3s-untreated HFD group. The protein expression of CAT and GPx in T3s-treated HFD mice tended to increase in the hippocampal region, but no significant differences were observed. These results indicate that treatment with T3s prevents brain oxidation via antioxidative effects as well as by upregulating antioxidative defense systems. However, the detailed mechanism remains to be elucidated. Further study is needed to clarify the mechanism controlling the upregulation of the T3s-induced antioxidative defense system.

The ratio of Akt and mTOR phosphorylation was determined in order to clarify the relationships between the Akt/mTOR pathway and oxidative stress in the obesity mice model (Figure 9). The ratio of Akt phosphorylation in the brains was enhanced by treatment with T3s and HFD compared to that of the CD group. Treatment with T3s enhanced the ratio of phospho-mTOR protein expression in the hippocampus of HFD-treated mice compared to that of the controls. mTOR regulates autophagy, protein translation, and stress resistance [9]. Considering that mTOR is inactivated via AMP-activated protein kinase (AMPK) and/or Akt pathway in the CR condition [9], we hypothesized that HFD may activate mTOR. However, the ratio of mTOR phosphorylation in the HFD group did not change. On the other hand, the ratio of mTOR phosphorylation in the T3s-treated HFD group was increased, and mTOR is phosphorylated by nutrients. Treatment with HFD may not be sufficient for mTOR phosphorylation, and additional factors may be needed for mTOR activation. Activated-mTOR, which inactivates the autophagy pathway, was increased in the hippocampus of T3s-treated HFD mice. This result indicates that the autophagy pathway may be inactivated in this model. Furthermore, it has been reported that the autophagy pathway is activated during neurodegeneration [23]. Neuronal function in the hippocampal region of T3s-treated HFD mice may be protected by the effect of T3s. However, further investigation is needed to clarify the relationship between changes in autophagy function and obesity. In addition, activation of Akt is involved in NF-E2 related factor 2 (Nrf2) phosphorylation. Nrf2 is degraded as a result of phosphorylation by glycogen synthase kinase 3 (GSK3)-β. GSK3-β is phosphorylated by Akt. The evidence shows that Akt phosphorylation induces antioxidative system upregulation [24,25]. The ratio of Akt phosphorylation in the hippocampus was significantly increased in the CD+T3s and HFD groups compared to that of the CD group. On the other hand, there were no changes in antioxidative enzyme expressions between the CD group and the other groups. However, the expressions of all antioxidative enzymes in the CD+T3s, HFD, and HFD+T3s groups tended to increase compared to that of the CD group. This phenomenon may be involved in Akt phosphorylation. Because there were no significant differences between the CD group and the other groups, the upregulation of antioxidative enzyme expressions were not only involved in Akt phosphorylation, and we have to check other pathways.

## 4. Materials and Methods 

### 4.1. Animals

All animal experiments were performed with the approval of the Animal Protection and Ethics Committee of Shibaura Institute of Technology (Tokyo, Japan) (approval code:18002, approval date: 20/06/2018). Nine-month-old male C57BL/6 mice were fed a HFD (#D12492; Research Diets Inc., New Brunswick, NJ, USA; 5.24 kcal/g, 60% of calories from fat) or control diet (CD) (#D12450; Research Diets Inc.; 3.58 kcal/g, 10% of calories from fat) in the presence or absence of T3s for 5 months. The nutrient composition of the CD and HFD is summarized in Table S1. Fifty milligrams of T3 mix (α:β:γ:δ = 33.2:4.2:46.1:15.0), kindly provided by Mitsubishi-Chemical Foods Corp. (Tokyo, Japan), was mixed with 100 g of CD (CD+T3s) or HFD (HFD+T3s). Animal housing conditions was kept constant (12-h light/dark cycle and a temperature of 24 ± 2 °C). The diets and water were provided to all animals with free access. The body weight and food intake were measured once per week, and the body weight gain and calorie intake were calculated from these data. After the treatment period, all mice cognitions were assessed using the cognitive trials. After assessments, all mice were sacrificed, and serum, liver, and brain (cerebrum cortex (Cortex), cerebellum, and hippocampus (Hippo) were collected for each analysis. 

### 4.2. Measurement of Cognitive Function

The cognitive function of all mice was assessed by the Rota rod (Muromachi Kikai Co., Ltd., Tokyo, Japan) and the Morris water maze tests, as described previously with some modifications [26,27,28]. The Rota rod was set to accelerate from 5 to 50 rpm in 120 s and the time and rpm required for the animal to fall from the Rota rod was measured. The trials were performed three times and were carried out every 20 min. 

The Morris water maze apparatus (Muromachi Kikai Co., Ltd.) (140 cm in diameter and 45 cm in height) consists of a pool constructed of acrylic resin. The bottom of the pool was divided into four quadrants by lines and had four different visible marks positioned around the pool. A submerged platform was placed in the center of one quadrant. The water temperature of the pool was maintained at 22 ± 2 °C. All mice were acclimated to experimenter handling prior to the experiment. The mice were allowed to swim freely for 60 s in the absence of platform and were handled over a 3 day period. Cognitive trials were performed 4 times per day and continued for 5 consecutive days. Escape latency (time to reach the goal), swimming distance, swimming speed, and the proportion of time spent swimming in the quadrant containing the platform were measured using the ANY-maze software (Stoelting Co., Wood Dale, IL, USA). The learning ability on each day was assessed by calculating the average escape latency of the four daily trials.

### 4.3. Measurement of Each Vitamin E Isoform

Each VE concentration was measured by high performance liquid chromatography (HPLC) with electrochemical detection (ECD), as described previously with some modifications [18]. Brain, serum, liver, and each diet were mixed with 6% pyrogallol (FUJIFILM Wako Pure Chemical Corp., Osaka, Japan) and 35% KOH solution (FUJIFILM Wako Pure Chemical Corp.), respectively. After mixing, a 2,2,5,7,8-Pentamethyl 6-chromanol (PMC) (Shigma Aldrich Corp., St Louis, MO, USA) solution was added as an internal standard, and then all samples were heated at 100 °C. After heating, all samples were cooled until room temperature (R/T). Then, a 1% NaCl solution and a mixed solution for VE extraction were added, and these mixtures were shaken. After centrifugation (3000 rpm and 4 °C), the upper layer was collected and evaporated. The residue was dissolved by methanol, and VE concentrations in this solution were measured using HPLC (Shiseido Co., Ltd., Tokyo, Japan).

### 4.4. Measurement of Antioxidative Enzyme Activities

The quantification of superoxide dismutase (SOD) activity was performed using a SOD Assay kit-WST (#S311; Dojindo Laboratory, Kumamoto, Japan) according to the manufacturer’s protocol. In this assay, SOD activity was measured using the decreasing ratio of WST-1 formazan. A microplate reader (#51119300, Multiskan GO; Thermo Fisher Scientific Inc., Hercules, CA, USA) was used for reading at 450 nm.

Glutathione peroxidase (GPx) activity was determined using a commercial kit (glutathione peroxidase cellular activity assay kit; Sigma-Aldrich), according to the manufacturer’s protocol. GPx activity was measured from the decreasing ratio of NADPH levels, which were measured at 340 nm absorbance every 10 sec for 1 min. 

Catalase (CAT) activity was determined by monitoring hydrogen peroxide levels, as described previously with some modifications [18]. Sample homogenates were mixed with 5 mM K-phosphate buffer, which was then rapidly incubated with hydrogen peroxide. The decreasing ratio of the hydrogen peroxide level was measured using a microplate reader (240 nm).

### 4.5. Western Blotting

Western blotting was done, as described previously with some modifications [18]. All samples were sonicated in lysis buffer and applied to Western blotting as described previously with some modifications [14]. The protein concentrations in samples were determined using a Bio-Rad protein assay (#500-006JA; Bio-Rad Japan, Tokyo, Japan) according to the manufacturer’s procedure. Protein samples (20 µg) were loaded on 15% sodium lauryl sulfate (SDS)-polyacrylamide gels and transferred to nitrocellulose membranes (FUJIFILM Wako Pure Chemical Corp.). The membranes were washed in TBS (Tris-HCl-buffered saline) containing 0.1% Tween-20 (TBS-T) and incubated in blocking solution (2 % skim milk in TBS-T) for 1 h at R/T. The membranes were treated with anti-SOD1 antibody (#ab13498; Abcam Plc., Cambridge, UK) at 1:2500, anti-GPx1 antibody (#ab22604; Abcam Plc.) at 1:4000, anti-CAT antibody (#ab195306; Abcam Plc.) at 1: 2000, mammalian target of rapamycin (mTOR) antibody (#2972; Cell Signaling Technology (CST) Inc., Danvers, MA, USA) at 1:1000, phospho-mTOR (Ser2448) antibody (#2971; CST Inc.) at 1:500, protein kinase B (Akt) (#9272; CST Inc.) at 1:4000, phospho-Akt (Ser473) antibody (#4060; CST Inc.) at 1:1000, or anti-α-tubulin antibody (#2125; CST Inc.) at 1:4000 dilution overnight at 4 °C. Anti-rabbit IgG HRP antibody (Promega Corp., Madison, WI, USA) was treated at 1:4000 dilution for 1 h at R/T. All chemiluminescent signals were generated by incubation with the detection reagents (Immobilon Western Chemiluminescent HRP substrate; Merck) according to the manufacturer’s procedure. The relative intensities were determined using the LAS-3000 system. The intensities of Ponceau S, SOD1, GPx1, CAT, mTOR, p-mTOR, Akt, and p-Akt were quantified using the ImageJ software (ImageJ 1.48v; National Institutes of Health, Bethesda, MD, USA).

### 4.6. Measurement of Lipid Peroxidation

To analyze brain oxidation, we measured thiobarbituric acid reactive substances (TBARS) using Yagi’s method, as described previously with some modifications [29]. The sample homogenate was mixed with 5 mM EDTA, 1% phosphoric acid, and 0.7% thiobarbituric acid. The mixture was heated to 100 °C for 45 min. After cooling, the samples were incubated with butanol for 3 min. Following centrifugation (3000 rpm, 10 min at 4 °C), the upper layer was isolated, and absorbance at 535 nm was read using a spectrophotometer (UV-1200; Shimadzu Corp., Kyoto, Japan).

### 4.7. Statistical Analysis

Data were plotted as mean ± SE of results of four independent experiments for each mouse group. Data were analyzed using the Tukey–Kramer’s method, with findings of *p* < 0.05 considered significant. In the case of the Morris water maze test, abnormal values were rejected based on the goal time on final-day trials (i.e., if the goal time of the final day was over 45 sec, the data of that mouse was rejected.).

## 5. Conclusions

In this study, we clarified that T3s delay body weight gain and investigated the possibility that T3s prevent brain oxidation. However, the detailed mechanisms have not yet been elucidated. We are continuing to study the relationship between obesity-related oxidative damage and the preventive effects of T3s.

## Supplementary Materials

Supplementary materials can be found at https://www.mdpi.com/1422-0067/21/12/4533/s1.
