# Supplementary material for: Tocotrienols Influence Body Weight Gain and Brain Protein Expression in Long-Term High-Fat Diet-Treated Mice
Source: Int J Mol Sci. 2020 Jun 25;21(12):4533. doi: 10.3390/ijms21124533 (PMC7352730; doi:10.3390/ijms21124533)
Supplement: Supplementary file 1 [file ijms-21-04533-s001.pdf]

International Journal of *Molecular Sciences*

MDPI

# Supplementary Material

# Tocotrienols Influence Body Weight Gain and Brain Protein Expression in Long-Term High-Fat Diet-Treated Mice

**Supplementary Table S1.** Nutrition components in CD and HFD

| | D12492 | | D12450J | |
|---|---|---|---|---|
| | g | kcal | g | kcal |
| Protein | 26 | 20 | 19.2 | 20 |
| Carbohydrate | 26 | 20 | 67.3 | 70 |
| Fat | 35 | 60 | 4.3 | 10 |
| Total | | 100 | | 100 |
| kcal/g | 5.24 | | 3.85 | |
| | **D12492** | | **D12450J** | |
| | g | kcal | g | kcal |
| Casein, 30 Mesh | 200 | 800 | 200 | 800 |
| L-Cystine | 3 | 12 | 3 | 12 |
| corn Starch | 0 | 0 | 506.2 | 2024.8 |
| Maltodextrin 10 | 125 | 500 | 125 | 500 |
| Sucrose | 68.8 | 275 | 68.8 | 275.2 |
| Cellulose, BW200 | 50 | 0 | 50 | 0 |
| Soybean Oil | 25 | 225 | 25 | 225 |
| Lard | 245 | 2205 | 20 | 180 |
| Mineral Mix S10026 | 10 | 0 | 10 | 0 |
| DiCalcium Phosphate | 13 | 0 | 13 | 0 |
| Calcium Carbonate | 5.5 | 0 | 5.5 | 0 |
| Potassium Citrate, 1 $H_2O$ | 16.5 | 0 | 16.5 | 0 |
| Vitamin Mix V10001 | 10 | 40 | 10 | 40 |
| Choline Bitartrate | 2 | 0 | 2 | 0 |
| FD&C Blue Dye #1 | 0.05 | 0 | | |
| FD&Yellow Dye #5 | | | 0.04 | 0 |
| Total | 773.85 | 4057 | 1055.05 | 4057 |
| **Mineral Mix S10026** | | | | |
| | g | Amt in 10g | | |
| Sodium Chloride | 259 | 1.0g | Na | |
| | | 1.6g | Cl | |
| Magnesium Oxide, Heavy, DC USP | 41.9 | 0.5g | Mg | |
| Magnesium Sulfate, Heptahydrate | 257.6 | 0.33g | S | |
| Ammonium Molybdrate Tetrahydrate | 0.3 | 1.6mg | Mo | |

| | | | |
|---|---|---|---|
| Chromium Posstasium Sulfate | 1.925 | 2.0mg | Cr |
| Copper Carbonate | 1.05 | 6.0mg | Cu |
| Ferric Citrate | 21 | 37mg | Fe |
| Manganase Carbqnate Hydrate | 12.25 | 59mg | Mn |
| Potassium Lodate | 0.035 | 0.2mg | I |
| Sodium Fluoride | 0.2 | 0.9mg | Fl |
| Sodium Sekenite | 0.035 | 0.16mg | Se |
| Zinc Carbonate | 5.6 | 29mg | Zn |
| Sucrose | 399.105 | 3.9911 | |
| Total | 1000 | | |
| **Vitamin Mix V10001** | | | |
| | g | Amt in 10g | |
| Vitamin A Acetate (500,000 IU/g) | 0.8 | 4000 IU | Vitamin A |
| Vitamin D3 (100,00 IU/g) | 1 | 1000 IU | Vitamin D3 |
| Vitamin E Acetate 500 IU/g | 10 | 50 IU | Vitamin E |
| Menadione Sodium Bisulfite 62.5% Menadione | 0.08 | 0.5 mg | Menadione |
| Biotine, 1.0% | 2 | 0.2 mg | Biotine |
| Cyanocobalamin, 0.1% | 1 | 10 µg | Vitamin B12 |
| Follic Acid | 0.2 | 2 mg | Follic acid |
| Nicotinnic Acid | 3 | 30 mg | Niacin |
| Calcium Pantothenate | 1.6 | 16 mg | Pantothenic Acid |
| Pyridoxine-HCl | 0.7 | 7 mg | Vitamin B6 |
| Riboflavin | 0.6 | 6 mg | Vitamin B2 |
| Thiamin-HCl | 0.6 | 6 mg | Vitamin B12 |
| Sucrose | 978.42 | | |
| Total | 1000 | | |

Vitamin E = dl- $\alpha$ -tocopherol acetate.
